# Editorial: Women in gastrointestinal and hepatic pharmacology 2022

**DOI:** 10.3389/fphar.2023.1359135

**Published:** 2024-01-08

**Authors:** Eleonora Lai, Jessica Williams, Raquel Abalo, Daniela Gabbia

**Affiliations:** ^1^ Medical Oncology Unit, University Hospital and University of Cagliari, Cagliari, Italy; ^2^ Department of Pharmacology and Toxicology, University of Kansas Medical Center, Kansas City, KS, United States; ^3^ Área de Farmacología y Nutrición, Departamento de Ciencias Básicas de la Salud, Universidad Rey Juan Carlos (URJC), Alcorcón, Spain; ^4^ Unidad Asociada R+D+i del Instituto de Química Médica (IQM), Consejo Superior de Investigaciones Científicas (CSIC), Madrid, Spain; ^5^ High Performance Research Group in Physiopathology and Pharmacology of the Digestive System (NeuGut-URJC). URJC, Alcorcón, Spain; ^6^ Grupo de Trabajo de Ciencias Básicas en Dolor y Analgesia de la Sociedad Española del Dolor (SED), Madrid, Spain; ^7^ Grupo de Trabajo de Cannabinoides de la SED, Madrid, Spain; ^8^ Department of Pharmaceutical and Pharmacological Sciences, University of Padova, Padova, Italy

**Keywords:** women in science, gastroenterology, hepatology, women in academia, pharmacology

The Research Topic “*Women in gastrointestinal and hepatic pharmacology*” was launched to highlight “the diversity of research performed across the entire breadth of Gastrointestinal and Hepatic Pharmacology” and to present “advances in theory, experiment, and methodology with applications to compelling problems” to promote gender equality in agreement with the United Nations Sustainable Development Goals (SDGs).

Until 1990, fewer than 5% of gastroenterologists were women (Feliu-Dominguez et al., 2017). Women representation has increased, but recent reports still show gender disparity favoring men in numbers (gastroenterology remains within the top 10 for male-dominated physician specialties; AMMC, 2021), salary, and leadership positions in both clinical and academic settings, irrespective of geographic area (Chua et al., 2021; Sethi et al., 2022; Kedia et al., 2023). Despite an early similar degree of interest as males, females perceive greater gender-based barriers to full development of a gastroenterology career as early as during their internal medicine residency (Advani et al., 2022). Barriers include family planning (such as concerns regarding fertility and radiation exposure, which impact subspecialties like advanced endoscopy), future work-life balance (stress, burnout), workplace hostility (less salary, less respect, unfair treatment), and perceived lack of female mentors/role models/sponsors leading to feeling “a lack of success,” which perpetuates further underrepresentation of women (Chua et al., 2021; Jamorabo et al., 2021; Advani et al., 2022; Rabinowitz, 2022).

Women are more prone to leave academic gastroenterology. Replacing faculty is both economically costly and a waste of scientific effort and talent (Waldman et al., 2004; Zimmermann, 2019; Tse et al., 2022). Thus, the scientific community is attracting and retaining female talent through different initiatives (Chua et al., 2021). For example, in 2014 the American Gastroenterological Association (AGA) initiated the Women’s Leadership Conference (WLC) to provide mentorship, teach skills and encourage engagement, leading to increased committee assignments and speaking opportunities for female gastroenterologists (Zimmermann, 2019). The World Gastroenterology Organization (WGO) implemented the Women in GI Webinar Series (WGO, 2023). The Equality and Diversity Plan of the United European Gastroenterology (UEG) includes measures to increase representation of women at all organization levels in all disciplines, functions, and contractual relations and highlights the need for integration of gender dimension in research activity, clinical guideline development and educational content (Esposito et al., 2023).

To counteract reduced visibility of females from senior publication authorship (Long et al., 2015), this Frontiers Research Topic required that the first or last author of submitted manuscripts be a researcher who identifies as a woman for consideration for publication. Of the 10 submissions received, 5 were accepted. As of today (9th December 2023), these manuscripts have accumulated 12 k views and 2,472 downloads. The collected papers are presented in the following paragraphs.

Chronic liver diseases of different etiologies may result in progression of fibrosis to cirrhosis with increased risk for hepatocellular carcinoma (HCC) development. For example, liver inflammation and fibrosis persist even after direct-acting antiviral (DAAs) treatment in some chronic hepatitis C virus (HCV) patients, leading to increased HCC risk. Cossiga et al. evaluated possible use of enhanced liver fibrosis (ELF) score as a non-invasive marker of fibrosis progression. ELF score was compared to transient elastography (TE) in a cohort of HCC patients before and after DAAs treatment. This study demonstrated that ELF score could represent an appropriate marker to be used in cases of TE unavailability or when reduced hospital access or prolonged physical contact are necessary, such as during the COVID-19 pandemic (Cossiga et al.).

The review by Lu et al. focused on main features and molecular mechanisms of ferroptosis in chronic liver diseases of different etiologies and therapeutic potential of compounds able to modulate this nonapoptotic cell death. This review discussed the effect of ferroptosis modulators in preclinical models as an overview of possible exploitation of ferroptosis for development of novel therapeutic approaches for chronic liver diseases (Lu et al.).

Non-alcoholic fatty liver disease (NAFLD) is a chronic metabolic disease characterized by hepatic steatosis, inflammation, fibrosis, and increased HCC risk and is a major health problem worldwide. Estrogen deficiency is associated with NAFLD in post-menopausal women. In their review, Zhao et al. assessed the protective role of phytoestrogens in post-menopausal NAFLD by discussing research evidence showing improvement in lipid and glucose metabolism, reduction of oxidative stress, inflammatory response, and fibrosis and regulation of intestinal microbiota (Zhao et al.). Reda et al. conducted preclinical research in NAFLD rats to assess the protective role of vitamin D3 against NAFLD and showed that Vitamin D3 supplementation induced improvement in serum biochemical markers, lipid profile, histopathological changes, antioxidant status, and modulation of inflammation and gene expression through SREBP-1-c/PPARα-NF-κB/IR-S2 signaling pathway.

Our Research Topic also includes a study on gastrointestinal cancer. The use of proton pump inhibitors (PPI) is very common for treatment. However, PPI-long-term use-induced hypergastrinemia can lead to proliferative modifications in gastrointestinal mucosa, which is a potential risk factor for gastric cancer (GC) and colorectal cancer (CRC), with controversial evidence. Guo et al. performed a systematic review and meta-analysis focused on GC and CRC case-control and cohort studies comparing PPI and non-PPI use. Authors demonstrated that PPI use and duration of administration was significantly associated with increased GC risk; conversely, no correlation with increased risk of CRC was found. These results suggest that indiscriminate and prolonged PPI use should be considered with caution (Guo et al.).

Despite challenges faced by female researchers in Gastroenterology and Hepatology, several tools may help them to achieve career success, including networking and joining professional societies (Kardashian and May, 2019; Tse et al., 2022). As mentioned above, gastroenterology societies (WGO, UEG, AGA, …) are doing an excellent job supporting women promotion. Contributing to special issues and Research Topics promoted by journals, such as this Frontiers initiative, is another impactful tool that female gastroenterologists should take advantage of.

As editors of the inaugural Frontiers “*Women in gastrointestinal and hepatic pharmacology*” article collection, we are thankful to all our contributing (female) authors and wish the next Frontiers article collection a great success, for the good of the specialty and, more importantly, of our (female) patients.

## References

[B1] AAMC (Association of American Medical Colleges) (2021). Active physicians by sex and specialty. Available at: https://www.aamc.org/data-reports/workforce/data/active-physicians-sex-specialty-2021 (Accessed December 9, 2023).

[B2] Advani R. Arjonilla M. Guerson A. Taub E. Monzur F. (2022). Gender-specific attitudes of internal medicine residents toward gastroenterology. Dig. Dis. Sci. 67 (11), 5044–5052. 10.1007/s10620-022-07541-5 35596822

[B3] Chua S. G. Wasan S. K. Long M. T. (2021). How to promote career advancement and gender equity for women in gastroenterology: a multifaceted approach. Gastroenterology 161 (3), 792–797. 10.1053/j.gastro.2021.06.057 34175283 PMC8403503

[B17] Esposito I. Simsek C. Nowak A. Tiniakos D. (2023). United European Gastroenterology Equality and Diversity. United European Gastroenterol J 11 (5), 484–487. 10.1002/ueg2.12415 PMC10256985 37209358

[B4] Feliu-Dominguez R. Medero-Rodriguez P. Cruz-Correa M. (2017). Women gastroenterologists in academic medicine: tradition versus transition. Dig. Dis. Sci. 62 (1), 13–15. 10.1007/s10620-016-4369-x 27832393

[B5] Gálvez-Robleño C. López-Tofiño Y. López-Gómez L. Bagüés A. Soto-Montenegro M. L. Abalo R. (2023). Radiographic assessment of the impact of sex and the circadian rhythm-dependent behaviour on gastrointestinal transit in the rat. Lab. Anim. 57 (3), 270–282. 10.1177/00236772221124381 36299170

[B6] Jamorabo D. S. Chen R. Gurm H. Jahangir M. Briggs W. M. Mohanty S. R. (2021). Women remain underrepresented in leadership positions in academic gastroenterology throughout the United States. Ann. Gastroenterol. 34 (3), 316–322. 10.20524/aog.2021.0597 33948055 PMC8079863

[B7] Kardashian A. May F. P. (2019). Empowering early career female gastroenterologists and hepatologists. Nat. Rev. Gastroenterol. Hepatol. 16 (11), 644–645. 10.1038/s41575-019-0216-9 31570808

[B8] Kedia D. Kamani L. Begum M. R. Nagral A. Mahtab M. A. Singh S. P. (2023). Journey of women in gastroenterology in south asian countries: from training to leadership. Euroasian J. Hepatogastroenterol 13 (1), 41–43. 10.5005/jp-journals-10018-1391 37554970 PMC10405808

[B9] Long M. T. Leszczynski A. Thompson K. D. Wasan S. K. Calderwood A. H. (2015). Female authorship in major academic gastroenterology journals: a look over 20 years. Gastrointest. Endosc. 81, 1440–1447. 10.1016/j.gie.2015.01.032 25887727

[B10] Rabinowitz L. G. (2022). Gender-specific attitudes of internal medicine residents toward careers in gastroenterology. Dig. Dis. Sci. 67 (11), 4969–4970. 10.1007/s10620-022-07550-4 35596821

[B11] Sethi S. Kumar A. Clough J. Ravindran S. Harris R. Harvey P. (2022). Women in gastroenterology: the UK trainee experience. Frontline Gastroenterol. 13 (6), 484–489. 10.1136/flgastro-2022-102101 36250176 PMC9555141

[B12] Tse C. S. Hinds S. Nguyen H. Xiong N. Moss S. F. Bhagra A. (2022). A 12-year north American longitudinal study of gender equity and equality in gastroenterology. Gastroenterology 162 (1), 63–67. 10.1053/j.gastro.2021.10.031 34743915

[B13] United Nations (2023). Departement of economic and social affairs, sustainable development, the 17 goals. Available at: https://sdgs.un.org/goals (Accessed December 9, 2023).

[B14] Waldman J. D. Kelly F. Arora S. Smith H. L. (2004). The shocking cost of turnover in health care. Health Care Manage Rev. 29, 2–7. 10.1097/00004010-200401000-00002 14992479

[B15] World Gastroenterology Organization (WGO) (2023). Women in gastroenterology webinars. Available at: https://www.worldgastroenterology.org/education-and-training/webinars/women-in-gi (Accessed December 9, 2023).

[B16] Zimmermann E. M. (2019). How to foster academic promotion and career advancement of women in gastroenterology. Gastroenterology 157 (3), 598–601. 10.1053/j.gastro.2019.07.041 31374216

